# DNA Methylation of the Angiotensinogen Gene, *AGT*, and the Aldosterone Synthase Gene, *CYP11B2* in Cardiovascular Diseases

**DOI:** 10.3390/ijms22094587

**Published:** 2021-04-27

**Authors:** Yoshimichi Takeda, Masashi Demura, Takashi Yoneda, Yoshiyu Takeda

**Affiliations:** 1 Department of Endocrinology and Metabolism, Kanazawa University Hospital, Kanazawa 920-8641, Japan; bell_bey@hotmil.com; 2 Department of Hygiene, Graduate School of Medical Science, Kanazawa University, Kanazawa 920-8641, Japan; m-demura@med.kanazawa-u.ac.jp; 3 Institute of Liberal Arts and Science, Kanazawa University, Kanazawa 920-8641, Japan; endocrin@med.kanazawa-u.ac.jp; 4 Endocrine and Diabetes Center, Asanogawa General Hospital, Kanazawa 920-8621, Japan

**Keywords:** angiotensinogen, aldosterone, methylation, cardiovascular disease, salt

## Abstract

Angiotensinogen (AGT) and aldosterone play key roles in the regulation of blood pressure and are implicated in the pathogenesis of cardiovascular diseases. DNA methylation typically acts to repress gene transcription. The aldosterone synthase gene *CYP11B2* is regulated by angiotensin II and potassium. DNA methylation negatively regulates *AGT* and *CYP11B2* expression and dynamically changes in response to continuous promoter stimulation of each gene. High salt intake and excess circulating aldosterone cause DNA demethylation around the CCAAT-enhancer-binding-protein (CEBP) sites of the *ATG* promoter region, thereby converting the phenotype of *AGT* expression from an inactive to an active state in visceral adipose tissue and heart. A close association exists between low DNA methylation at CEBP-binding sites and increased *AGT* expression in salt-sensitive hypertensive rats. Salt-dependent hypertension may be partially affected by increased cardiac *AGT* expression. CpG dinucleotides in the *CYP11B2* promoter are hypomethylated in aldosterone-producing adenomas. Methylation of recognition sequences of transcription factors, including CREB1, NGFIB (NR4A1), and NURR1 (NR4A2) diminish their DNA-binding activity. The methylated CpG-binding protein MECP2 interacts directly with the methylated *CYP11B2* promoter. Low salt intake and angiotensin II infusion lead to upregulation of *CYP11B2* expression and DNA hypomethylation in the adrenal gland. Treatment with the angiotensin II type 1 receptor antagonist decreases *CYP11B2* expression and leads to DNA hypermethylation. A close association between low DNA methylation and increased *CYP11B2* expression are seen in the hearts of patients with hypertrophic cardiomyopathy. These results indicate that epigenetic regulation of both *AGT* and *CYP11B2* contribute to the pathogenesis of cardiovascular diseases.

## 1. Introduction

**Figure 1:**
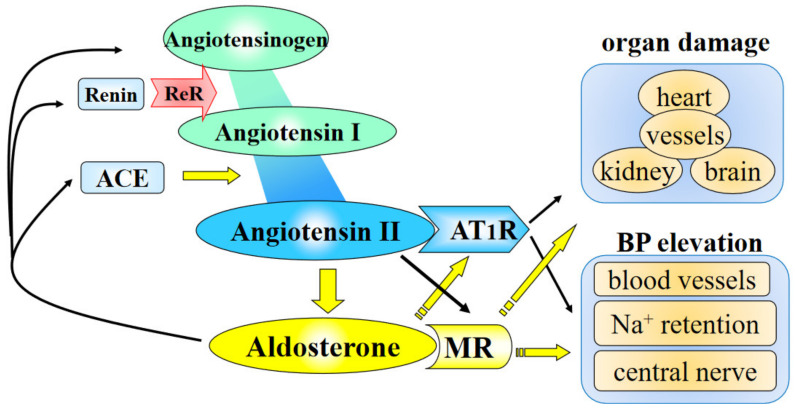
The cross-talk of angiotensin II and aldosterone in the pathogenesis of hypertension and cardiovascular diseases. Angiotensin II increases MR activity. Aldosterone affects not only AT1R but also ACE, renin and angiotensinogen. AT1R, angiotensin II type 1 receptor; MR, mineralocorticoid receptor; ACE, angiotensin converting enzyme.

The renin–angiotensin–aldosterone system (RAAS) plays an important role in the pathogenesis of cardiovascular diseases [1,2,3]. Angiotensinogen (AGT) is the only known substrate for renin that is the rate-limiting enzyme of the RAAS. AGT levels can control the activity of the RAS, and its upregulation may lead to elevated angiotensin peptide levels and increases in blood pressure (BP) and is implicated in cardiovascular injuries. The cross-talk of angiotensin II and aldosterone in the pathogenesis of hypertension and cardiovascular diseases has been proposed (Figure 1). Aldosterone affects not only the type1 angiotensin II receptor (AT1R) but also the angiotensin converting enzyme (ACE) and the renin receptor [4,5,6,7,8].

Hypertensive mice that systemically express human renin and human AGT show elevated renal angiotensin II levels [9]. We have reported that renal AGT gene expression is increased in salt-sensitive hypertensive rats and treatment with the AT1R blocker (ARB) or mineralocorticoid receptor antagonist (MRA) decreases the AGT mRNA levels [10]. Recent studies on experimental animal models and transgenic mice have documented the involvement of the adipose AGT in the activation of the RAAS and the development of hypertension. This observation indicates that the adipose tissue-derived AGT contributes to circulating the AGT level and the resultant pathophysiology of obesity-related metabolic diseases [11,12,13].

Aldosterone is produced in the zona glomerulosa of the adrenal cortex by aldosterone synthase (CYP11B2) and is known to promote cardiac fibrosis and hypertrophy with concurrent elevation of inflammatory and oxidant signaling [14,15,16,17]. Aldosterone synthesis is regulated by angiotensin II and potassium. Patients with primary aldosteronism, have a higher incidence of myocardial infarction and stroke than do patients with essential hypertension [18,19,20,21,22,23]. Mineralocorticoid receptor antagonists (MRAs) have been shown to be effective in reducing the mortality and hospitalizations for heart failure (HF) in patients with chronic HF with a reduced left-ventricular ejection fraction (rEF) [24,25], and in patients with HFrEF early postmyocardial infarction [26,27,28]. Experimental animal data support a role for aldosterone in mediating cardiovascular and renal injuries. In salt-sensitive hypertensive (SSH) rats, administration of the MRA greatly attenuated cardiac hypertrophy [29]. An important pathological effect of aldosterone in the heart has also been reported in experimental models of mineralocorticoid hypertension [30]. In these studies, prolonged exposure to aldosterone was associated with the development of myocardial hypertrophy and fibrosis.

DNA methylation is a stable, long-term epigenetic modification that affects gene expression without altering its DNA sequence [31,32]. In mammals, DNA methylation is a covalent modification that occurs primarily at a position on cytosine rings within CpG dinucleotides that usually represses gene transcription. Additionally, DNA hypermethylation in gene promoter regions generally silences genes. DNA methylation has a central role in the development and differentiation of mammalian cells. A logical connection between carcinogenesis and DNA methylation has been well established. Somatic DNA changes and dysregulation of DNA methylation have recently been demonstrated to be involved in the development of hypertension [33]. It is hypothesized that once DNA methylation patterns are established during cellular development, differentiation and disease progression, they are stably maintained. However, recent progress in this field has revealed DNA methylation pattern dynamics in response to various environmental stimuli [34,35]. Daily factors involved in the alteration of DNA methylation include chemicals, infection, smoking, exercise and learning. Lifestyle influences the metabolism via DNA methylation. These observations imply an association between DNA methylation dynamics and multifactorial lifestyle-related diseases, such as cardiovascular diseases, diabetes mellitus and dyslipidemia.

## 2. Contribution of DNA Methylation to Human AGT Gene Transcription

**Figure 2:**
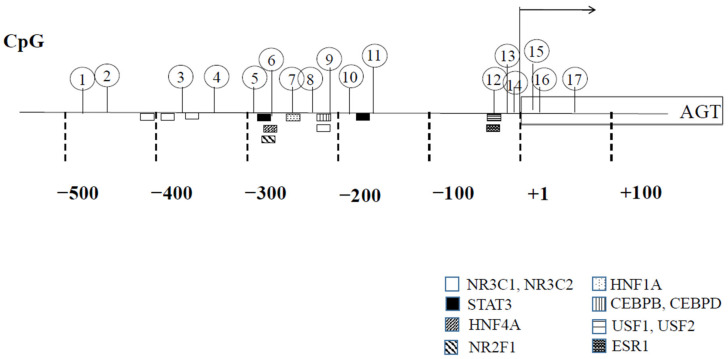
CpG dinucleotides and transcription factor-binding sites in the *AGT* promoter. Nucleotide numbers are relative to the transcription start site. CpG dinucleotides from −483 to + 78 are numbered from the 5′ to 3′ ends.

DNA methylation is generally involved in stabilizing the silent state of genes by either blocking DNA-binding transcription factors or recruiting methyl-CpG-binding domain (MBD) proteins, which favor the formation of the transcriptionally inactive forms of chromatin (heterochromatin). Among the MBD proteins, methyl-CpG-binding protein 2 (MECP2), MBD1 and MBD2 repress the transcription of methylated gene promoters. The human *AGT* promoter possesses a number of CpG dinucleotides that are targets for DNA methylation (Figure 2). The human *AGT* promoter which is located within a CCAAT enhancer-binding protein (CEBP)-binding site that contains a CpG dinucleotide at positions −218/−217, is hypomethylated in tissues and cells with high *AGT* expression (liver, heart and HepG2 hepatocytes) but not in those with low expression (adrenal glands, leukocytes and adrenocortical H295R cells) [16]. Thus, the methylation status of a CpG dinucleotide within the CEBP-binding site appears to be inversely associated with *AGT* expression. In the human *ATG* promoter, each of these sites, upstream stimulatory factor (USF)1/estrogen receptor (ESR)1, CEBPB/nuclear receptor (NR)3C1, hepatocyte nuclear factor (HNF)1A, 4A, USF1,2, NR2F1 and ESR1 contains each CpG dinucleotide (Figure 2).

Overall, 17 out of the 18 CpG dinucleotides within the human *AGT* promoter and its first exon (−459 to +66) have been analyzed to determine where and how DNA demethylation and remethylation affects the interleukin 6 (IL6) stimulation. DNA demethylation was detected within 12 h of IL6 stimulation and reached a maximum level 24 h following stimulation. The first DNA demethylation event occurred around a CEBP-binding site (positions −225/−217) as well as a transcription start site (TSS); DNA demethylation from CpG9 to CpG12 had started by that time, spreading out and stretching to CpG2 on the 5′-upstream side and to CpG17 on the 3′-downstream side during IL6 stimulation [36].

Local DNA demethylation is one of several steps in the serial recruitment of multiple transcription factors. In addition to CEBP, the signal transducer and activation transcription factor 3 (STAT3) and HNF1A are involved in the IL6-induced *AGT* transcription in human hepatocytes. The positive effect of STAT3 on the IL6-induced *AGT* transcription is mediated by its interaction with its two target DNA sites (positions −278/−269 and positions −171/−162). IL6 also induces the interaction between HNF1A and the *AGT* promoter at positions −247/−236.27. Additional transcription factors, such as STAT3 and HNF1A, are recruited when chromatin is relaxed and DNA is unmethylated, as MBD proteins have difficulty interacting with DNA without methylated CpG dinucleotides. DNA demethylation on the 5′-upstream side reaches CpG2 (position −434/−433), suggesting that as-yet-unidentified DNA-binding transcription factors are also involved in the IL6-induced *AGT* transcription. Following the IL6 withdrawal, DNA remethylation concurrently progresses in a reverse manner over the next 24 h [36].

## 3. Effect of Salt Intake on Methylation Status of the AGT Gene in the Heart

Excess sodium intake is intimately involved in the pathogenesis of hypertension [37,38,39,40,41]. In large populations, significant correlations between the level of salt intake, BP, and the frequency of hypertension have been reported [42]. Several studies have shown that high salt intake reduces circulating RAAS but increased tissue RAAS in salt-sensitive hypertensive (SSH) rats [43,44]. Human aldosterone synthase-transgenic mice are reported to be SSH [45]. Tissue AGT is known as an important effector system for the regulation of BP. The overexpression of the AGT gene in the heart increases BP and cardiac hypertrophy and young spontaneously hypertensive rats show an elevation of tissue AGT expression [46]. High salt intake increases the cardiac AGT as well as the AT1R mRNA levels in SSH rats [29]. Treatment with MRA decreases the tissue AGT and improves cardiovascular injuries independent of the blood pressure [47].

**Figure 3:**
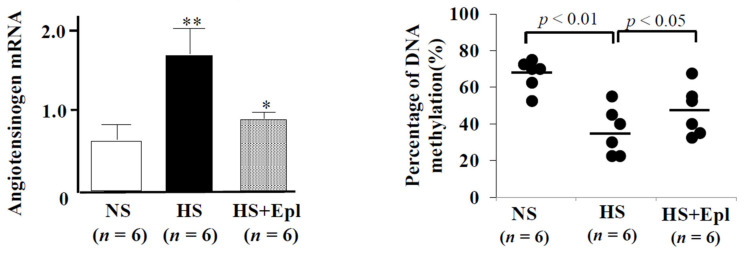
Effects of a high-salt diet on *ATG* mRNA expression and methylation of *ATG* in the heart of SSH rats. A low-salt diet significantly increased the *ATG* mRNA levels and decreased the methylation ratio of *ATG*. Treatment with eplerenone decreased the *ATG* mRNA levels and increased the methylation ratio of *ATG*. NS, normal-salt diet; HS, high-salt diet; HS + Epl. High-salt diet with treatment with eplerenone. ** *p* < 0.01 vs. NS; * *p* < 0.05 vs. HS.

CEBP has a pivotal role in the transcriptional regulation of the *AGT* gene in both humans and rats [16]. Two CEBP-binding sites (CTTGCTCCA, positions +2 to +10; and CTGGGAA, positions +78 to +84) exist in the first exon of the rat *AGT* gene. High salt intake increases the *AGT* mRNA levels in the heart and demethylates the *AGT* promoter in SSH rats (Figure 3). Treatment with eplerenone decreases the AGT mRNA levels and methylates the *AGT* promoter in SSH rats. DNA demethylation occurs around the TSS and these CEBP-binding sites. These results suggest that a salt-associated stimulatory signal may recruit CEBP to its binding sites in the first exon to activate *AGT* transcription. Treatment with MRA decreased *AGT* expression and increased the methylation ratio in SSH rats. This is an interesting finding since MRA influences epigenetics and causes beneficial effects to cardiovascular diseases. Based on the role of epigenesis in the development of chronic cardiovascular and metabolic diseases, it is presumed that epigenetic intervention may be an effective strategy for the treatment of these diseases. Wang et al. [48] reported the increased expression and promoter hypomethylation of the *AT1R* gene in the adipose tissue of high-fat-fed spontaneously hypertensive rats were reversed by treatment with losartan. Epigenetic drugs, such as DNA methyltransferase inhibitors, were investigated in animal models of cardiovascular diseases [49]. These drugs had a deleterious effect. MRA as well as ARB may serve as a potential inducer of epigenetic modification without a deleterious complication.

## 4. Epigenetic Modification of AGT Gene in Aldosterone-Producing Adenoma

Primary aldosteronism (PA) has been reported with increasing frequency and accounts for 5–10% of the hypertensive population [50]. Increased prevalence of both diabetes mellitus and metabolic syndrome in patients with PA has been reported in the German Conn’s Registry and in the Japan Primary Aldosteronism Study (JPAS) [51,52]. Wu et al. [53] reported that the expression of genes related to fibrosis and adipogenesis in adipose tissue was higher in patients with aldosterone-producing adenoma (APA) than in patients with non-functioning adenoma (NFA). Kalupahara et al. [12] reported that overproduction of *AGT* from adipose tissues induces adipose inflammation, glucose intolerance, and insulin resistance.

Wang et al. [36] reported the differential effects of cortisol and aldosterone on AGT transcription in visceral adipose tissue. An excess of aldosterone in APA elicited low DNA methylation levels at all CpG dinucleotides from CpG1 to CpG13 in the *AGT* promoter with an accompanying upregulation of adipose tissue *AGT* expression. However, an excess of cortisol in Cushing’s adenoma did not affect the methylation status of *AGT*. They observed dynamic changes in DNA methylation patterns following IL6 stimulation, the first DNA demethylation event occurred around CpG9 in the AGT promoter. CpG9 exists in a DNA sequence that functions as both a GRE and a CEBP-binding site (Figure 2), implying that the activation of MRs (NR3C2) but not GRs (NR3C1) initiates chromatin remodeling along with CEBP. Members of the NR3C subfamily and many other nuclear receptor superfamily members act cooperatively with other DNA-binding transcription factors. The interaction of their target DNA sequences can integrate the hormonal response to other regulatory pathways. As is the case with the IL6-induced *AGT* activation, MRs and other transcription factors, such as CEBP, STAT3, and HNF1A, can bind DNA between CpG1 and CpG13 of the *AGT* promoter and might be involved in the transcriptional activation of *AGT* when there is an excess of aldosterone. Collectively, these results suggest that activated MRs bind DNA at positions −225/−217 and have a leading role in the transcriptional activation of *AGT*, thereby recruiting other transcription factors.

## 5. Contribution of DNA Methylation to Human *CYP11B2* Transcription

Aldosterone biosynthesis is under the control of potassium and angiotensin II, which increase the levels of *CYP 11B2* mRNA and lead to an increase in the activity of aldosterone synthase [54,55,56,57,58]. High potassium diets in rats increase the expression of *CYP11B2* in the adrenal gland and aldosterone production [59].

**Figure 4:**
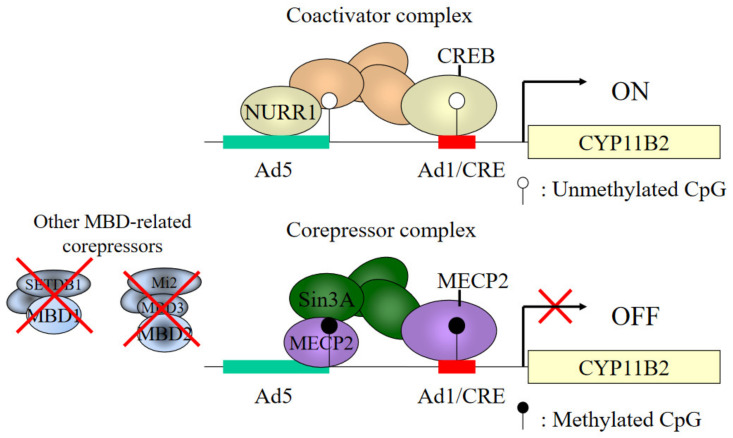
Epigenetic changes of the *CYP11B2* promoter region. Binding activities of coactivator complex, such as CREB and NURR1 and corepressor complex MECP2 are regulated by DNA methylation. Methylation of Cpg1 greatly decreased the CREB1 binding to Ad1. DNA methylation at CpG2 reduced the basal binding activities of NR4A1(NGF1B) and NR4A2 (NURR1) with A5. DNA methylation increased the MECP2 binding to CpG1 and CpG2. The closer relationship between other MBD-related corepressors and gene expression should be clarified.

A number of *cis*-acting regulatory elements have been identified in the *CYP11B2* promoter: Ad1 (CRE, cAMP response element), Ad4, Ad5 and NBRE-1 (NGFI-B (nerve growth factor-induced clone B) response element). ATF1 or CREB1 bind to Ad1/CRE, leading to increased *CYP11B2* transcription (Figure 4). Potassium as well as Ang II have the ability to activate these transcription factors [11,60,61].

**Figure 5:**
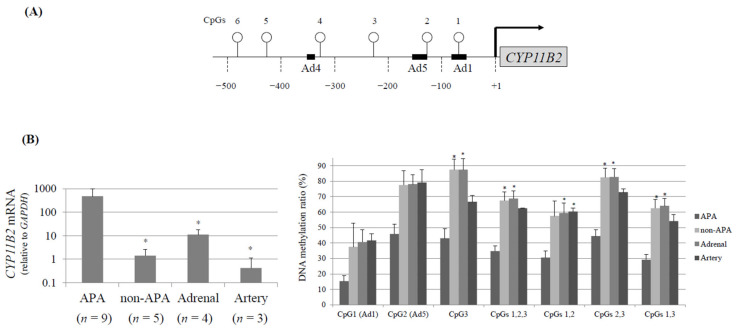
(**A**) Schema of CpG dinucleotides within the human *CYP11B2* gene promoter. Open circle denotes CpG dinucleotides. (**B**) Inverse correlation between DNA methylation and *CYP11B2* mRNA levels. The *CYP11B2* mRNA level was significantly elevated in APA. The CpG methylation ratios of the *CYP11B2* promoter was significantly decreased in APA. * *p* < 0.05 compared to aldosterone-producing adenoma (APA) (*n* = 9, APA; *n* = 5, non-APA; *n* = 4, the adrenal gland; *n* = 3, the artery).

Figure 5A shows the CpG sites of the *CYP11B2* promoter region. Transcriptional activation by potassium gradually causes DNA demethylation around the CREB1- and NR4A-binding sites as well as a TSS over seven days, and its withdrawal reverses this process over the next seven days in H295R cells [62]. Although the CYP11B2 promoter is a non-CpG island promoter, each of the CREB1- and NR4A-binding sites (at positions −71/ −64 and positions −129/−114, respectively) contains one CpG dinucleotide, and these binding sites are crucial for the basal transcription activity. In addition, two CpG dinucleotides (positions +37/+38, +45/+46) are located near the TSS. This CpG content of the *CYP11B2* promoter offers a much greater opportunity to precisely analyze the association between DNA methylation pattern dynamics, transcription factor recruitment and chromatin relaxation than the *AGT* promoter. As is the case with the *AGT* promoter, DNA demethylation occurs upon stimulation, whereas remethylation occurs when the stimulus is removed within the *CYP11B2* promoter. Changes in DNA demethylation at the NR4A -binding site and around the TSS occur two days later than those at the CREB1-binding site. The NR4A-binding site, its TSS and the CREB1-binding site are remethylated to pretreatment levels 2, 4 and 7 days following the end of stimulation, respectively. These changes in DNA methylation patterns are linked to both chromatin relaxation as well as the abundance of transcription factor interactions [34]. Collectively, dynamic changes in DNA methylation following stimulation occur in relaxed chromatin regions, both where transcription factors actively interact and where transcription is initiated.

We have reported that methylation of CpG1 greatly decreased the CREB1 binding to Ad1 (21). DNA methylation at CpG2 reduced the basal binding activities of NR4A1 (NGF1B) and NR4A2 (NURR1) with Ad5 by 30% and 50%, respectively [63]. Previous work has shown that high immunoreactivity of both NGF1B and NURR1 in the zona glomerulosa of the adrenal gland [64]. The pattern of CpG methylation and expression levels of NGF1B and NURR1 appear to have a synergistic effect on the *CYP11B2* transcription in the adrenal cortex.

## 6. Epigenetic Regulation of *CYP11B2* by Angiotensin II and Salt Intake

In the adrenal cortex, the expression of the aldosterone synthase gene, *CYP11B2*, is mainly controlled by the body sodium status via the RAS. Sodium restriction or Ang II infusion significantly increases *CYP11B2* mRNA levels in the rat adrenal gland [65]. We reported that angiotensin II infusion in rats decreased the methylation ratio of *CYP11B2* and increased gene expression in the adrenal gland [63]. A low salt diet induces hypomethylation of the rat *CYP11B2* and increases the *CYP11B2* mRNA levels parallel with the aldosterone synthesis. Plasma renin activity is also increased by a low salt diet. These results clearly indicate the influence of the angiotensin II in the epigenesis of *CYP11B2* in the adrenal gland. Nishimoto et al. [66] reported that the rat zona glomerulosa transcriptome was changed by the dietary sodium intake, involving more than 280 differentially regulated genes. Gene ontology analysis identified three different gene groups; cell proliferation, response to a stimulus, and cholesterol/steroid metabolism. They suggested that the transcriptome changes were caused not only by the activation of the RAS but also by the neurological responses.

Aldosterone breakthrough is a phenomenon in which plasma aldosterone concentrations increase above pretreated levels after a long-term therapy with angiotensin-converting enzyme (ACE) inhibitors or Ang. II type 1 receptor blocker (ARB) [67,68,69,70,71]. This phenomenon may have important clinical consequences because the increased aldosterone in a high salt state may facilitate cardiovascular and renal damage in hypertensive patients [72]. The involvement of various in vivo factors, such as ACTH, electrolytes, endothelins, and Ang II type 2 receptor actions [39] have been proposed to explain this breakthrough phenomenon; however, the detailed underlying mechanism remains unknown. Hashimoto et al. [47] reported that the direct renin inhibitor as well as ARB caused the aldosterone breakthrough without plasma endothelin elevation. Long-term treatment with ACEI or ARB may cause CpG demethylation of CpGs 1 and 2 in the adrenal cortex, leading to aldosterone breakthrough.

## 7. Epigenetic Modification of *CYP11B2* in Aldosterone-Producing Adenoma

Hypomethylation of *CYP11B2* was detected in APAs and a negative correlation between the CYP11B2 mRNA levels and the methylation ratio was seen in our clinical samples (Figure 5B). Hypomethylation of *CYP11B2* may be an important regulatory mechanism of gene expression not only in the normal adrenal gland but also in APA [73]. Howard et al. [74] reported that a CpG island in the promoter region of *CYP11B2* was hypomethylated in APAs but not in blood DNA from the same patients. Yoshii et al. [75] reported the methylation rate in CpG (−116), CpG (−471) and CpG (−757) were lowered in APAs compared with non-functioning tumors, but no significant correlation between methylation rates and mRNA levels was seen. They also demonstrated that a somatic KCNJ5 mutation did not influence the methylation rate in APAs. Nishimoto et al. [76] reported an interesting case of a patient in whom nodule development from subcapsular aldosterone-producing cell clusters (APCCs) caused hyperaldosteronism. The APCCs lie beneath the adrenal capsule and like APA, many APCCs harbor somatic gene mutations are known to increase the aldosterone production [77,78]. These findings suggest that APCCs may play a role in pathologic progression of PA. However, Omata et al. [79] reported that APCC are frequent in non-hypertensive Japanese adrenals, accumulate with age and frequently harbor somatic mutations (most commonly in the calcium voltage-gated channel subunit alpha1 D (*CACNA1D*)). We found that the *KCNJ5* mutation was detected in aldosterone-producing microadenoma and APCC in which hypomethylation of *CYP11B2* was seen (unpublished data). Sun et al. [80] demonstrated specific subgroups of APCC with strikingly divergent distribution patterns of metabolites. The relationship between steroid metabolomics and the genome and epigenome of *CYP11B2* in APCC should be clarified.

Medical treatment with a MRA, such as spironolactone, eplerenone or esaxerenone, is recommended to patients with PA due to bilateral adrenal disease or patients with unilateral PA who are unable or unwilling to undergo surgery [81,82]. Several papers reported cases of idiopathic hyperaldosteronism with spontaneous remission after potassium canrenoate therapy [83,84,85]. Yoneda et al. [86] reported a case of APA with remission after long-term spironolactone therapy. Ye et al. [87] reported that spironolactone inhibited basal and angiotensin II stimulated aldosterone synthesis in human adrenal cells. These data suggest the possibility of spironolactone acting on the methylation of *CYP11B2* in both normal and aldosterone-producing adenoma cells.

## 8. Gene Expression and CpG Methylation of *CYP11B2* in Tissues from Cardiomyopathy

Hypertrophic cardiomyopathy (HCM) is the most common genetic heart disease, characterized by complex pathophysiology, heterogenous morphology, and variable clinical manifestations. The main goals of pharmacological therapy in HCM include the treatment of the left-ventricular dysfunction and HF and the control of the atrial fibrillation (AF) [88,89,90,91,92]. Huang et al. [93] reported that a lower risk of new AF is observed in HCM patients treated with ACE inhibitors or ARBs compared with those receiving neither of these medications. MRAs are reported to be effective for prevention of AF [94].

**Figure 6:**
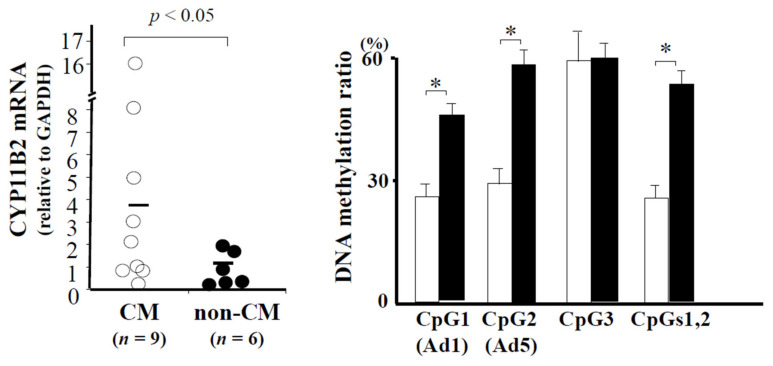
The *CYP11B2* mRNA level was significantly increased in the heart of HCM. The CpG methylation ratios of the *CYP11B2* promoter was significantly decreased in the heart of HCM. * *p* < 0.05 compared to non-HCM.

**Figure 7:**
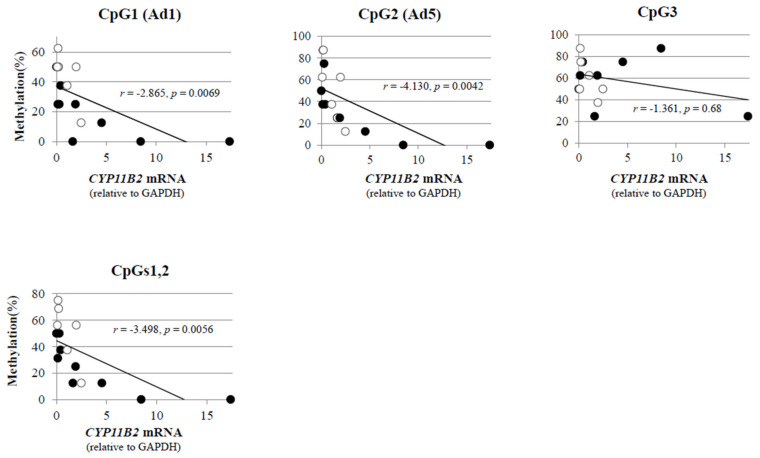
Inverse correlation between the CpG methylation ratios and the *CYP11B2* mRNA levels. Statistically significant nonparametric inverse correlations were observed between the CpG methylation ratio and the *CYP11B2* mRNA level. The CpG methylation at CpGs 1 and 2, but not CpG3, was inversely correlated with the *CYP11B2* mRNA levels in the heart.

Aldosterone production and *CYP11B2* gene expression have been shown to be upregulated in cardiac tissues during hypertrophic cardiomyopathy (HCM), and these are recognized as one of the major modifiers of the HCM phenotype [95]. We analyzed CpG methylation ratios and CYP11B2 mRNA levels using simultaneously isolated DNA and RNA from both HCM (*n* = 9) and non-HCM (*n* = 6) tissues. The CYP11B2 mRNA levels of HCM were 3.9-fold higher than those of non-HCM (Figure 6). HCM tissues were hypomethylated at CpGs 1 and 2 compared to non-HCM tissues. The methylation ratios of CpGs 1 and 2 in HCM were approximately half of those in non-HCM (Figure 6). Inverse significant nonparametric correlations were observed between the CYP11B2 mRNA levels and the methylation ratios of CpGs 1 and 2 (Figure 7). These results indicated that increased CYP11B2 mRNA levels were associated with CYP11B2 demethylation in HCM and that CYP11B2 mRNA levels were inversely correlated with CYP11B2 methylation in human myocardium.

Aldosterone has direct effects on cardiac hypertrophy and fibrosis [10,96,97]. Aldosterone is locally produced in cardiovascular tissues and makes an impact via paracrine or intracrine mechanisms [98,99,100]. Garnier et al. [101] reported that transgenic mice overexpressing *CYP11B2* in the heart showed a coronary endothelium-independent dysfunction. Alesutan et al. [102] reported that the vascular *CYP11B2* contributes to the stimulation of the vascular smooth muscle cells osteo-/chondrogenic transformation during hyperphosphatemia. Aldosterone synthesis in human myocardium in acute myocarditis is reported [103]. The observed increased myocardial *CYP11B2* mRNA levels in patients with HCM is in accordance with the results of previous studies showing cardiovascular aldosterone synthesis. A clear association between CpG methylation and *CYP11B2* gene expression in the heart is provided. We predict that DNA methylation at CpGs 1 and 2 is a key determinant of the *CYP11B2* mRNA levels in the heart, and that hypomethylation of the *CYP11B2* promoter causes an aberrant increase in the *CYP11B2* gene expression in the pathophysiology of cardiac hypertrophy, heart failure, or cardiomyopathy. The molecular mechanisms regulating the demethylation of CpGs1 and 2 in the heart in general, and in HCM in particular, remain to be established.

Mutations in more than 30 different genes have been found to cause inherited HCM, with some been associated with a very poor prognosis [90]. However, the contribution of the causal mutations to phenotype severity can be relatively modest, with the modifier genes playing a significant role [104,105]. Identification of the modifier genes will complement the results of causative gene studies and could enhance genetic-based diagnosis, risk stratification, and implementation of preventive and therapeutic measures in patients with HCM. Epigenomic changes cause an aberrant activation of the *CYP11B2* in HCM patients and they shed light on the underlying epigenomic mechanisms that enhance the severity of the cardiac phenotype.

## 9. Epigenesis of Angiotensin Converting Enzyme Gene

The angiotensin-converting enzyme (ACE) plays a central role in the RAAS, which is involved in the pathogenesis of cardiovascular diseases [106,107,108]. We have reported increased *ACE* gene expression in the heart and kidney of SSH [10,29]. Epigenetic regulation of somatic ACE by DNA methylation and histone acetylation is reported [109]. Inhibition of DNA methylation stimulates ACE mRNA expression. Mudersbach et al. [110] reported that DNA methylation determined the tumor necrosis factor (TNF)a-dependent regulation of the ACE gene transcription and thus protein expression in human endothelial cells. Lam et al. [111] reported that ACE genetic variants influenced methylation and modified the association between depression and methylation. They also reported that ACE methylation was a suitable biomarker of the cortisol and/or hypothalamic–pituitary–adrenal (HPA) axis. Activation of the HPA axis induces the glucocorticoid and mineralocorticoid receptor activity and is implicated in cardiovascular diseases [112]. Further studies are necessary to determine whether methylation of the ACE gene influences the glucocorticoid or mineralocorticoid receptor activity.

## 10. Conclusions

The presence of local RAAS in cardiovascular and adipose tissues and their influence in cardiovascular and metabolic diseases are described. Gene expression of *AGT* and *CYP11B2* is reversibly regulated by an epigenetic modification. Both a high salt diet and an excess aldosterone production influence the methylation status of the *AGT* and *CYP11B2* genes. The relationship between the epigenetic regulation of RAAS by environmental factors or hormone excess and the pathogenesis of cardiovascular diseases should be further clarified.

## Data Availability

Not applicable.

## Abbreviations

ACE	angiotensin converting enzyme
Ad	cis-acting element
AGT	angiotensinogen
AMP	adenylic acid
Ang II	angiotensin II;
ARB	AT1R blocker;
APA	aldosterone-producing adenoma
APCC	aldosterone-producing cell cluster
ATF	activating transcription factor
AT1R	type2 angiotensin II receptor
BP	blood pressure
CACNA1D	Calcium Voltage-Gated Channel Subunit Alpha1 D
CEBP	CCAAT enhancer binding protein
CREB	cyclic AMP responsive element binding protein
CYP11B2	aldosterone synthase
Epl	eprelenone
ESR	estrogen receptor
GR	glucocorticoid receptor
GRE	GR element
HCM	hypertrophic cardiomyopathy
HF	heart failure
HNF1A	hepatocyte nuclear factor1 homeobox A
HPA	hypothalamic-pituitary-adrenal
IL 6	interleukin 6
JPAS	Japan primary aldosteronism study
KCNJ	potassium inwardly rectifying channel J subfamily J
MBD	methyl-CpG-binding domain
MECP	methyl-CpG-binding protein
Mi2	chromodomain-helicase-DNA-binding protein Mi-2 homolog
MR	mineralocorticoid receptor
MRA	mineralocorticoid receptor antagonist
NGFI-B	nerve growth factor-induced clone B
NBRE-1	NGFI-B response element
NFA	non-functioning adenoma
NR4A	nuclear receptor 4 group A
NS	normal salt
NURR1	nuclear receptor-related factor 1
PA	primary aldosteronism
RAAS	renin-angiotensin-aldosterone system
rEF	reduced left-ventricular ejection fraction
ReR	renin receptor
SETDB	histone-lysine N-methyltransferase
Sin3A	SIN3 transcription regulator family member A
SSH	salt-sensitive hypertension
STAT	signal transducer and activation transcription factor
TNF	tumor necrosis factor
TSS	transcription start site
USF	upstream stimulatory factor
